# Global Tumor RNA Expression in Early Establishment of Experimental Tumor Growth and Related Angiogenesis following Cox-Inhibition Evaluated by Microarray Analysis

**Published:** 2007-05-01

**Authors:** Hans Axelsson, Christina Lönnroth, Marianne Andersson, Wenhua Wang, Kent Lundholm

**Affiliations:** Department of Surgery, Surgical Metabolic Research Laboratory at Lundberg Laboratory for Cancer Research, Sahlgrenska University Hospital, Göteborg, Sweden

**Keywords:** RNA expression, microarray, angiogenesis, cyclooxygenase

## Abstract

Altered expression of COX-2 and overproduction of prostaglandins, particularly prostaglandin E_2_, are common in malignant tumors. Consequently, non-steroidal anti-inflammatory drugs (NSAIDs) attenuate tumor net growth, tumor related cachexia, improve appetite and prolong survival. We have also reported that COX-inhibition (indomethacin) interfered with early onset of tumor endothelial cell growth, tumor cell proliferation and apoptosis. It is however still unclear whether such effects are restricted to metabolic alterations closely related to eicosanoid pathways and corresponding regulators, or whether a whole variety of gene products are involved both up- and downstream effects of eicosanoids. Therefore, present experiments were performed by the use of an in vivo, intravital chamber technique, where micro-tumor growth and related angiogenesis were analyzed by microarray to evaluate for changes in global RNA expression caused by indomethacin treatment.

Indomethacin up-regulated 351 and down-regulated 1852 genes significantly (p < 0.01); 1066 of these genes had unknown biological function. Genes with altered expression occurred on all chromosomes.

Our results demonstrate that indomethacin altered expression of a large number of genes distributed among a variety of processes in the carcinogenic progression involving angiogenesis, apoptosis, cell-cycling, cell adhesion, inflammation as well as fatty acid metabolism and proteolysis. It remains a challenge to distinguish primary key alterations from secondary adaptive changes in transcription of genes altered by cyclooxygenase inhibition.

## Introduction

It is well established that non-steroidal anti-inflammatory drugs (NSAIDs) attenuate tumor net growth (Peterson, 1986), where provision of unselective prostaglandin synthase inhibitors, particularly indomethacin, attenuates systemic inflammation due to reduced prostaglandin production. This leads to decreased host appearance of acute phase proteins in both tumor-bearing mice and cancer patients (Cahlin et al. 2000b; Peluffo et al. 2004; McMillan et al. 1995). Such effects are related to reduced tumor growth, improved appetite and attenuation of cachexia with subsequent prolongation of survival in experimental models (Gelin et al. 1991; Cahlin et al. 2000a; Lönnroth et al. 1995). Similar improvements have also been repeatedly observed in unselected cancer patients on systemic anti-inflammatory treatment (Lundholm et al. 1994; Lundholm et al. 2004). In a recent study we reported that unspecific COX-inhibition (indomethacin) interfered with early onset of tumor endothelial cell growth, tumor cell proliferation and apoptosis (Lönnroth et al. 1995), observations that are similar to our findings in prostaglandin receptor subtype knock out mice (Axelsson et al. 2005). It is however still unclear whether such effects are mainly restricted to metabolic alterations closely related to eicosanoid and prostanoid pathways or whether a whole variety of gene products are involved up- and downstream of effects by eicosanoids. Therefore, microarray technique was used to evaluate overall changes in global RNA expression in early onset of micro-tumor growth and related angiogenesis.

## Material and Methods

### Tumor models and animal groups

#### Tumor model

A methylcholanthrene induced sarcoma (MCG 101) was used in present experiments. This tumor model has been continuously transplanted in vivo at our laboratory for more than 25 years. The tumor was originally induced chemically as a sarcoma, while recent histological evaluation revealed that few tumor cells, if any, have characteristics of a sarcoma. Therefore, our tumor should rather be classified as a low or undifferentiated rapidly growing epithelial-like solid tumor. It has a reproducible and exponential growth pattern with a doubling time of 55–60 hours in vivo. It leads to 100% tumor take and does not give rise to visible metastases within the time period it kills the host. The tumors comprise 15–20 % of the body weight of the tumor bearing animals at the time of spontaneous death due to anorexia and cachexia. MCG 101 cells produce increased systemic levels of prostaglandin E_2,_ and COX-1/COX-2 inhibition by indomethacin, with normalized systemic levels of PGE_2_, reduced tumor growth, improved nutritional state and prolonged host survival (Gelin et al. 1991; Cahlin et al. 2000a). These effects by indomethacin were in part due to decreased tumor cell proliferation and increased apoptosis as well as attenuated angiogenesis (Axelsson et al. 2005).

MCG-101 tumor cells for intravital chamber inoculation were maintained in Mc Coy’s 5 A medium (MP Biomedicals, Inc., Aurora, Ohio, USA) supplemented with fetal calf serum (FCS, 10%), penicillin (100 U/ml), streptomycin (100 μg/ml) and L-glutamine (292 μg/ml). Cells were split 1/5 once weekly with a medium change in between (Mc Coy’s 5A + 2% FCS, penicillin, streptomycin and L-glutamin as mentioned above). The viability of the tumor cells was >99 % evaluated by trypan blue exclusion and microscopic examination before experiment. At start of experiments, cells were trypsinized and suspended in Mc Coy’s 5A medium at a concentration of 1.15 × 10^5^ cells/μl, 0,5 μl was inoculated into the intravital chamber as described. Animals were sacrificed at day 5 and micro tumors were immediately frozen in liquid nitrogen and kept at −80 °C until RNA extraction.

#### Animal groups

Adult, weight stable, female, wild type C57 black mice (n = 24) were used. The animals were housed in a temperature controlled room (24 °C) with a 12 hour light / dark schedule. The mice were housed in separate cages during the experiments to avoid interference with the subcutaneously placed intravital chamber. All animals were allowed initial adaptation for 3 days following operation to attain stable body weight and normal food intake before experiments started with free access to ordinary rodent chow (ALAB AB, Stockholm, Sweden) and water ad libitum under all experimental conditions.

All mice were randomly assigned to treatment (n = 12) and control groups (n = 12) before implantation with tumor cell suspensions. Treatment groups received indomethacin (Confortid, 5 mg/ ml, Dumex-Alpharma) provided in the drinking water for 5 days corresponding to 6 μg / ml drinking water (Gelin et al. 1991; Cahlin et al. 2000a; Lönnroth et al. 1995; Lönnroth et al. 2001). The appropriate dilution of indomethacin in the drinking water was calculated based on daily normal water consumptions of 3–4 ml water/ mouse/day. This corresponds to indomethacin of 1 μg/g bw/day. Controls received ordinary drinking water. Indomethacin provision started two days before tumor cell inoculation.

### Intravital chamber and surgical techniques

The dorsal skin fold chamber in the mouse was prepared as described earlier (Axelsson et al. 1997). Mice were anaesthetized with i.p. injection of 0.15 ml from a 1 ml stock-solution composed of 0.4 ml Ketalar^®^ (50 mg/ml; Parke-Davis), 0.05 ml Rompun^®^ vet (20 mg/ml; Bayer), and 0.55 ml physiological saline. The dorsal skin was shaved. The mice were kept at constant temperature of 36–37 °C by means of a heating pad during the procedure. A 20 mm long midline incision was made in the dorsal skin. Blunt dissection was used to free the skin from underlying tissue before introducing the chamber into the skin fold. The chamber was fixed to the skin by 4–0 sutures and the midline incision used for the installation of the chamber was closed with two 4–0 stitches. The skin covering one side of the central hole of the chamber was removed to expose the micro vascular tree of the contralateral subcutaneous tissue. Tumor cells (5.75 × 10^4^) were inoculated onto the upper tissue layer of the chamber preparation by means of a Hamilton needle (10 μl). Small volumes of tumor cells were applied in order to avoid disseminated growth of tumor in the chamber. The access chamber was closed by cover glass after inoculation.

### Microscopy

Observations on tumor growth were made by microscopy in a Nikon Eclipse E400 microscope with Nikon Plan 4X/0,10 objective and Nikon Digital Camera DXM 1200. Photographical documentations were performed immediately after implantation of tumor cells at day 0 and at day 5 following tumor cell implantation. Digital pictures were kept in a computer for subsequent analysis. Typical tumor graphs have been published elsewhere (Axelsson et al. 2005; Axelsson et al. 1997).

### Image analysis of vascular beds

Image analyses were based on analysis of a given area by use of a digital photo across the tumor area. The area was composed of the tumor and its near surroundings and was the identical area in photos from day 0 and day 5. The centrum of a photo corresponded to the central part of the tumor based on visual analysis. For image analyses we used the computer program Easy Image Analysis 2000, Tekno Optik AB, and applied a technique to quantify the area (mm^2^) of tumor related blood vessels and the size of the tumor area in the same plane (mm^2^). Tumor related vascular area is the difference in vascular area between day 5 and day 0. This represents the appearance of net vessel formation around the tumor during five days, which was reported to be significantly reduced by indomethacin(Axelsson et al. 2005). Five days tumor growth was necessary to identify microtumors and corresponding vascular net works for isolation of RNA.

### RNA extraction and amplification

Tumors from experiment 1 with 6 indomethacin-treated mice and 5 untreated control mice were pooled within groups, 3 + 3 (Indo 1A, 1B) and 3 + 2 (Ctrl 1C, 1D). Tumors from experiment 2 with 6 indomethacin-treated and 6 control mice were pooled within groups in the same way, (Indo 2A, 2B and Ctrl 2C, 2D). Total RNA Isolation Microdissected Cryosections Kit (QIAGEN Sciences, Maryland, U.S.A.) was used. Tissue disruption was done by aspiration with a syringe through 18 gauge needle 5x in lysis buffer. Quality and quantity of RNA were checked in an Agilent 2100 BioAnalyzer with RNA 6000 Nano Assay kit (Agilent Technologies, Palo Alto, CA, U.S.A.). Concentrations of RNA were measured in a NanoDrop (ND-1000A) spectrophotometer (NanoDrop Technologies, Inc.). Isolated tumor weight ranged from 8.4 to 16 mg (Indo) and 7.2 to 20.1 mg (Ctrl) wet weight and total RNA amount ranged from 4.1 to 10.1 μg (booth groups). RNA was linearly amplified with BD Smart mRNA Amplification Kit (BD Biosciences Clontech, Palo Alto, CA, USA). Unamplified total RNA used in the amplification reaction ranged from 425 ng to 946 ng with an efficiency of 160 to 240 x amplification based on the assumption that 5% of the total RNA fraction consists of polyA + mRNA. Amplified mRNA was checked for quality and quantity as mentioned above for total RNA.

### cDNA Microarray profiling and data analysis

Expression array, Whole Mouse Genome Oligo Microarray (Agilent Technologies), containing 44290 features, including positive and negative control spots, was used. Four-hundred nanograms of amplified mRNA fractions from indomethacin-treated animals in experiment 1 (pool of 200 ng 1A and 200 ng 1B = test) were labeled with Cyanine 3-dCTP (Amersham Biosciences) in cDNA synthesis reaction with Agilent Fluorescent Direct Label Kit. Fourhundred nanograms of amplified mRNA fractions from untreated control mice in experiment 1 (pool of 200 ng 1C and 200 ng 1D = ctrl) were labeled with Cyanine 5-dCTP in parallel with the test-fraction. Hybridizations were performed during 18 hours with test- versus control cDNA followed by post-hybridization washes according to “in situ Hybridization Kit Plus” (Agilent Technologies) instructions. Microarrays were dried with nitrogen gas in a laminar flow bench and images were quantified on Agilent G2565 AA microarray scanner and fluorescence intensities were extracted using the Feature Extraction software program (Agilent technologies). Dye-normalized, outlier- and background-subtracted values were further analyzed in Gene-Spring software program, imported with the FE Plug-in (Agilent Technologies). Amplified mRNAs from experiment 2 were analyzed in the same way as in experiment 1 as a replicate. Technical replicates of experiment 1 and 2 were performed in a second run and the computer-based analyses were performed on all four series.

Gene expression in healthy, inbred mice was tested in muscle tissue from two individuals for assessment of the overall microarray variability. PolyA + selected RNA was extracted and 400 ng from mouse 1 were labeled with Cyanine 3-dCTP and 400 ng from mouse 2 were labeled with Cyanine 5-dCTP followed by hybridization to the same array targets with a ratio of 1.31 ± 0.03 (M ± SD) which confirms the validity of presented findings.

### Statistics

Comparisons between several groups were performed by factorial analysis of variance (ANOVA). The ratio between expressed transcripts in tumor tissue of MCG 101 inoculates from study versus control animals were calculated in the GeneSpring software program. Genes with p-values outside the 99% confidence limit (p < 0.01) of the overall analytical variability as derived by t-testing were regarded to reflect significantly up- or down-regulated genes as defined by the software producer.

## Results

24 mice were used in these experiments divided on 12 study animals and 12 controls. One mouse in the control group died initially due to the experimental procedures subsequently to implantation of the vascular chamber and was excluded from further analyses.

**Figure 1.**
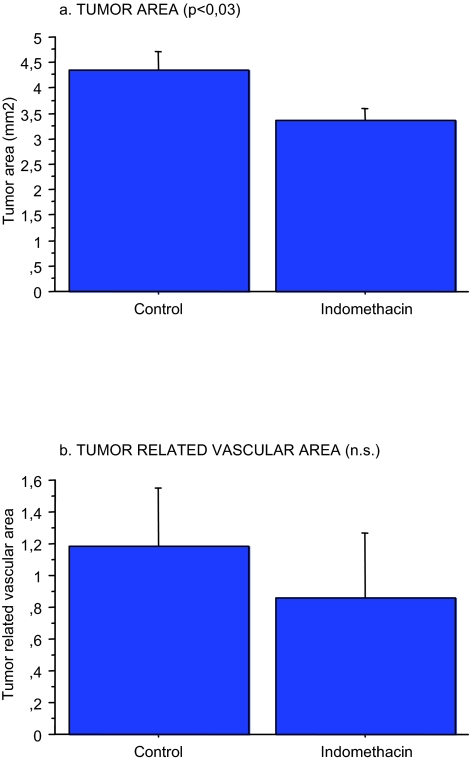
Effects on early tumor growth (a) (p < 0.03) and tumor related vascular area (b) by indomethacin provision in the drinking water to MCG 101, inoculated mice (n = 12) compare to controls (n = 12) as described in Methods.

Indomethacin treatment reduced tumor area (p < 0.03), while the reduced tumor related vascular area did not reach statistical significance as observed and reported elsewhere in a larger group of animals (Axelsson et al. 2005) (Fig. 1). Tumor vascular area is positively correlated to the tumor area (not shown) as reported (Axelsson et al. 2005).

**Figure 2.**
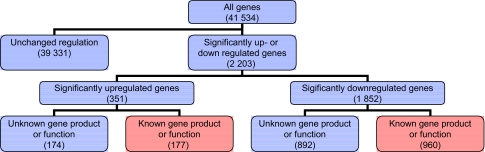
Stepwise analysis of global gene expression assessed by microarray technique. Genes in red boxes are subjected to functional considerations in Figure 4 and Table 4–6.

**Figure 3.**
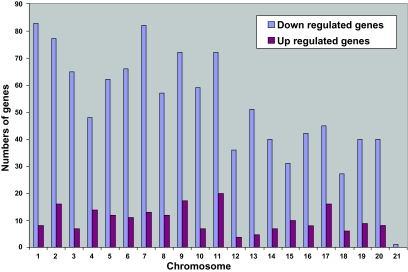
Distribution of tumor tissue genome wide expression of RNA transcripts (genes) significantly up- and down-regulated during indomethacin treatment of MCG 101 inoculated mice. (p < 0.01) (141 of the up-regulated genes and 756 of the down-regulated genes had unknown localization).

**Figure 4.**
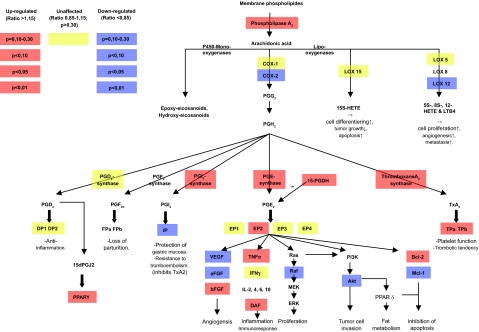
Alterations in overall tumor tissue levels of transcript expression in arachidonic acid metabolism during indomethacin treatment of MCG 101 inoculated mice. Blue boxes indicate down-regulated genes and colors turning towards red indicate increasing grades of up-regulation of transcripts.

41 534 transcripts (genes) were analyzed (Fig. 2). Indomethacin treatment up-regulated 351 and down-regulated 1852 genes (p < 0,01). 1066 of these 2203 genes had unknown gene products and unknown biological function(s) of the corresponding protein. Such genes were therefore excluded for further consideration (Fig. 2). Genes significantly affected by indomethacin were located on all chromosomes as shown in (Fig. 3) with distribution according to functional aspects as shown in Table 1. The ten most up- and down-regulated genes according to the amounts of transcript following indomethacin treatment are shown in Table 2. Genes altered by indomethacin and strictly related to arachidonic acid metabolism are shown in Figure 4. Significantly altered genes for regulation of important but different aspects of the carcinogenic process (inflammation, angiogenesis, apoptosis, cell cycle, proliferation, cell adhesion, carbohydrate and fatty acid metabolism, proteolysis) are shown in the Appendix ordered by their magnitude in transcription comparing indomethacin treated tumors versus controls.

## Discussion

It is well-recognized that non-steroidal anti-inflammatory drugs (NSAIDs), particularly indomethacin, attenuate tumor net growth (Peterson 1986), reduced tumor related cachexia, improved appetite and prolonged survival in tumor bearing mice (Gelin et al. 1991; Cahlin et al. 2000a; Lönnroth et al. 1995), and in part also in cancer patients (Lundholm et al. 1994; Lundholm et al. 2004). There is also evidence from population based, case control and clinical trials that regular use of NSAIDs may reduce the relative risk to develop colorectal adenomas (Baron et al. 2003; Sandler et al. 2003) and colorectal cancer (Smalley and DuBois 1997; Thun et al. 1991; Williams et al. 1999; Giovannucci et al. 1994; Giovannucci et al. 1995; Smalley et al. 1999). Altered expression of COX-2 and overproduction of prostaglandins are common in colorectal cancers (Eberhart et al. 1994; Marnett and DuBois 2002; Williams et al. 1999; Joo et al. 2002), breast (Rolland et al. 1980), gastric (Uefuji et al. 2001), esophagus (Shamma et al. 2000), pancreatic (Juuti et al. 2006), bile duct (Hayashi et al. 2001), papillary thyroid (Siironen et al. 2006), malignant pheochromocytomas (Salmenkivi et al. 2001), urinary tract (Tuna et al. 2004), prostate (Tkacz et al. 2005), cervical (Kulkarni et al. 2001), retinoblastoma (Karim et al. 2000) and lung cancer (Wolff et al. 1998). Elevated levels of COX-2 and PGE_2_ are also seen in premalignant conditions such as Barrett′s esophagus (Kandil et al. 2001) and colorectal adenomas (Khan et al. 2001). Thus, there are strong evidence that both COX-1 and COX-2 are of importance in several cancer forms in man. (Gupta et al. 2003; Huang et al. 2006).

We have earlier reported that indomethacin increased tumor cell apoptosis and reduced tumor cell proliferation and endothelial cell growth in animal studies (Axelsson et al. 2005), where unselective prostaglandin synthase inhibitors, (indomethacin), attenuate systemic inflammation reflected by reduction in plasma concentrations of prostaglandin E_2_ and acute phase proteins (Cahlin et al. 2000b; Peluffo et al. 2004; McMillan et al. 1995). It is assumed that tumor reducing effects by indomethacin are caused by blocking prostaglandin production (Vane 1971), with competitive inhibition of substrate binding at COX- isoenzymes. Highly selective COX-2 inhibitors have retained anti-tumor effects, despite a lack of COX-1 inhibition, suggesting that COX-2 mediators are as well important for tumor development (Sheng et al. 1997; Evans 2003; Peluffo et al. 2004; Huang et al. 2006). The present study was based on intravital chamber (Axelsson et al. 1997) and microarray technique in order to evaluate overall changes in global RNA synthesis caused by indomethacin treatment on initial tumor growth to further map out important genetic areas behind tumor reducing effects by cyclooxygenase inhibition.

Indomethacin reduced tumor size as in earlier studies (Axelsson et al. 2005) and altered expression of 2 203 genes out of 41 534 (5,3%). These genes were widely and relatively uniformly spread over the entire genome on all chromosomes. Indomethacin down-regulated five times as many genes being up-regulated. Affected genes were predominantly stimulatory in function. Thus, effects by indomethacin were overall down-regulating stimulatory genes. However, indomethacin influenced on the expression of a large number of genes responsible for important steps in the carcinogenic process. Genes with mainly reduced expression involved apoptosis, cell cycle and proliferation, cell adhesion, fatty acid metabolism and proteolysis, while effects on angiogenesis, carbohydrate metabolism and inflammation displayed both up-and down-regulation.

We have previously reported that tumor effects by indomethacin involve apoptosis and cell proliferation in vivo (Axelsson et al. 2005), as confirmed by others in vitro (Huang et al. 2006). Earlier and resent studies indicate that COX-2 derived PGE_2_ are involved in the control of programmed cell death (Sheng et al. 1998; Huang et al. 2006), although exact mechanisms are unknown. PGE_2_ may reduce apoptotic rates by increasing levels of antiapoptotic proteins like Bcl-2 (Sheng et al. 1998), Mcl-1 or other key mediators as NF-κB (Poligone and Baldwin 2001) and by activation of two main apoptotic pathways: the death receptor and mitochondrial pathways (Huang et al. 2006). Such results agree with our present in vivo findings, which indicate that indomethacin down-regulated Mcl-1, while Bcl-2 and TNF-receptor associated factor 5 were significantly increased. Genes involved in the NF-κB cascade were both up- and down-regulated by indomethacin. Prior studies have shown that PGE_2_ stimulates cell proliferation in colorectal cancer (Sheng et al. 2001), although downstream pathways are still unknown. There are indications that cell proliferation is stimulated by activation of the epidermal growth factor receptor (EGFR) (Yoshimoto et al. 2002). Tumor growth is also stimulated by the oncogene Ras, which induces cell proliferation, cell transformation and cell survival by activation of Raf-MEK-ERK and PI3K-Akt pathways. Indomethacin treatment in present experiments down-regulated Raf and Akt genes, which would reduce tumor cell proliferation. Genes responsible for the production of EGF and EGF-receptor were not significantly changed, while other genes involving cell proliferation mainly showed reduced expression as reported elsewhere (Lönnroth et al. 2001).

Angiogenesis is though to play a central role in cancer progression (Folkman 1971). Main proangiogenic factors are vascular endothelial growth factor (VEGF) and fibroblast growth factor 1 & 2 (acidFGF & basicFGF). There is also evidence that COX-2 plays a role in tumor-associated angiogenesis (Williams et al. 2000; Oshima et al. 1996) by modulation of proangiogenic factors with correlations between COX-2 and VEGF expression in tumor tissue (Joo et al. 2003). PGE_2_ is then thought to be the mediator behind COX-2 activities in tumor angiogenesis (Hernandez et al. 2001). Both selective and nonselective COX-inhibitors can however reduce tumor angiogenesis, by inhibiting production of proangiogenic factors and subsequent proliferation, migration and tube formation of endothelial cells (Axelsson et al. 2005; Peterson 1986; Tsujii et al. 1998; Masferrer et al. 2000; Skopinska-Rozewska et al. 1998; Sawaoka et al. 1999). In the present analysis the gene coding for one of three forms of VEGF (VEGF-A) was down-regulated by indomethacin whereas the other two (VEGF-B & C) were unaffected. AcidFGF showed a trend towards down-regulation, while basicFGF displayed a trend to up-regulation. Other genes in angiogenesis were mainly down-regulated. Our results therefore support that indomethacin affects tumor angiogenesis in addition to other processes more related to tumor cell proliferation (Axelsson et al. 2005).

Malignant disease is also characterized by attenuation of cell mediated anti-tumor immune response. Probably directed in part by PGE_2,_ based on reduced production of anti-tumor Th1 cytokines (TNFα, IFNγ and IL-2) (Harris et al. 2002) and increased production of Th2 cytokines (IL-4, IL-10 and IL-6) (Shreedhar et al. 1998; Huang et al. 1998; Della Bella et al. 1997). In present experiments the gene coding for TNFα was up-regulated, while genes coding for mentioned cytokines were not changed by indomethacin. Our previous results imply that main tumor reducing effects by indomethacin are caused by blockade of prostaglandin production (Vane 1971). Accordingly, genes coding for these enzymes in prostaglandin biosynthesis were decreased by indomethacin including the COX-2 enzyme. This suggests that indomethacin also acts on transcription of cyclooxygenases.

Genes in control of fatty acid and protein metabolism were significantly and highly down-regulated by indomethacin, while genes for carbohydrate metabolism seemed to be both up- and down-regulated. These observations may contribute to reported beneficial overall host-metabolic effects by indomethacin attenuating catabolism caused by a growing tumor (Gelin et al. 1991; Lundholm et al. 2004). Distant metastases are the major cause of death in cancer. Over-expression of COX-2 and increased production of PGE_2_ may promote metastatic potential and tumor cell invasiveness (Tsujii et al. 1997), while treatment with NSAIDs reduced this imbalance (Lundholm et al. 1994; Yamauchi et al. 2003; Yao et al. 2003). The PI3K-Akt pathway is highly important in regulating tumor cell migration and invasion where PGE_2_ is known to stimulate this pathway (Sheng et al. 2001). In present experiments the gene coding for Akt was significantly down-regulated and genes coding for proteins regulating cell adhesion were likewise mainly down-regulated by indomethacin.

In conclusion, this study has evaluated the magnitude of net overall changes on whole genome transcription in micro-tumors during a period of unspecific cyclooxygenase blockade by indomethacin. The results, derived in vivo by application of microarray, demonstrate an overwhelming number of influences by indomethacin of which inhibitory effects (down-regulation) appeared to dominate. It remains a challenge to distinguish primary key effects from secondary adaptive alterations in transcription of all these affected genes by NSAID treatment.

## Figures and Tables

**Table 1.** The number of up- and down-regulated genes, regarded either stimulatory or inhibitory in function for progression of microtumors and related angiogenesis.

	**Up-regulated**	**Down-regulated**	**P-value (sign test)**
**Angiogenesis**	1	4	n.s.
Stimulatory genes	1	3	n.s.
Inhibitory genes	0	1	n.s.
**Apoptosis**	9	22	0.05
Stimulatory genes	9	19	n.s.
Inhibitory genes	0	3	n.s.
**Carbohydrate metabolism**	4	5	n.s.
Stimulatory genes	4	5	n.s.
Inhibitory genes	0	0	n.s.
**Cell cycle & cell proliferation**	8	39	<0.01
Stimulatory genes	7	31	<0.01
Inhibitory genes	1	6	n.s.
**Cell adhesion**	3	19	<0.01
Stimulatory genes	3	19	<0.01
Inhibitory genes	0	0	n.s.
**Fatty acid metabolism**	3	16	<0.01
Stimulatory genes	3	16	0.01
Inhibitory genes	0	0	n.s.
**Inflammation**	9	14	n.s.
Stimulatory genes	8	13	n.s.
Inhibitory genes	1	1	n.s.
**Proteolysis**	5	26	<0.01
Stimulatory genes	5	26	<0.01
Inhibitory genes	0	0	n.s

**Table 2.**
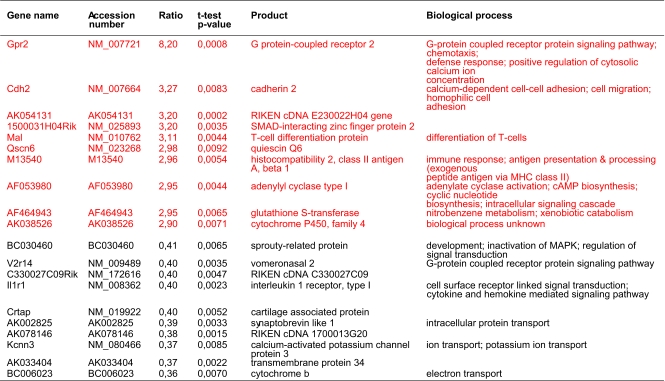
Ten most up- and down-regulated tumor tissue genes during indomethacin treatment of MCG 101 inoculated mice.

The 99% confidence interval for the relative expression ratio was 1.31±0.03 (2.3%) based on 4 arrays.

**Table:**
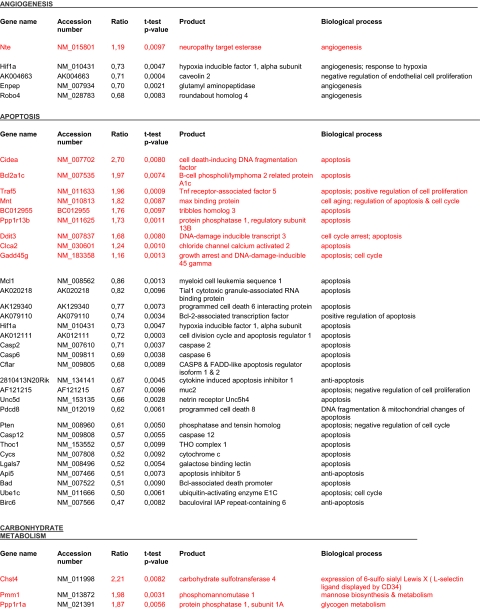

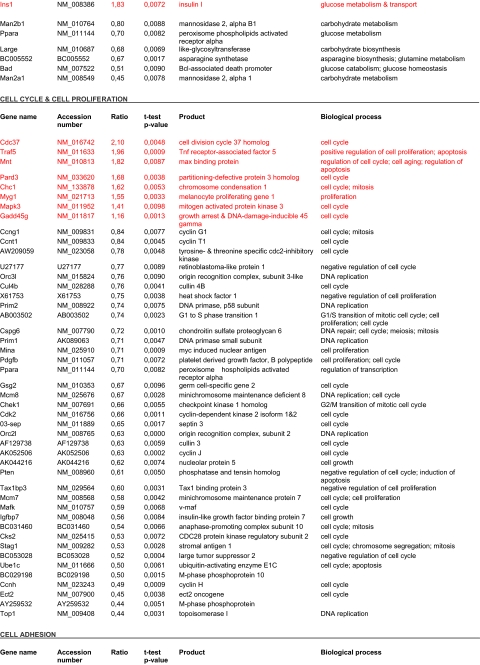

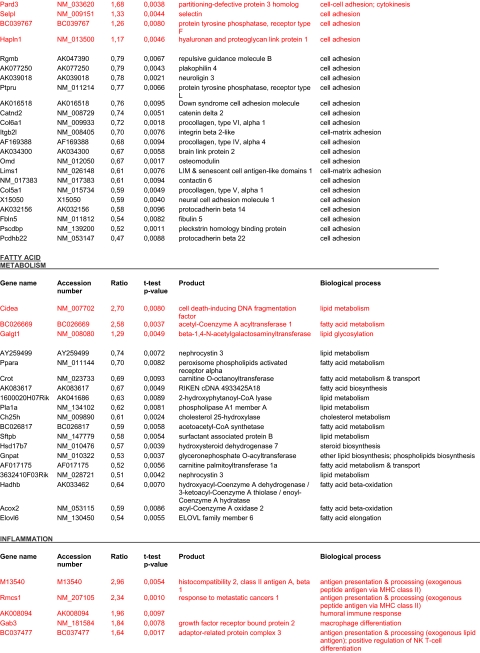

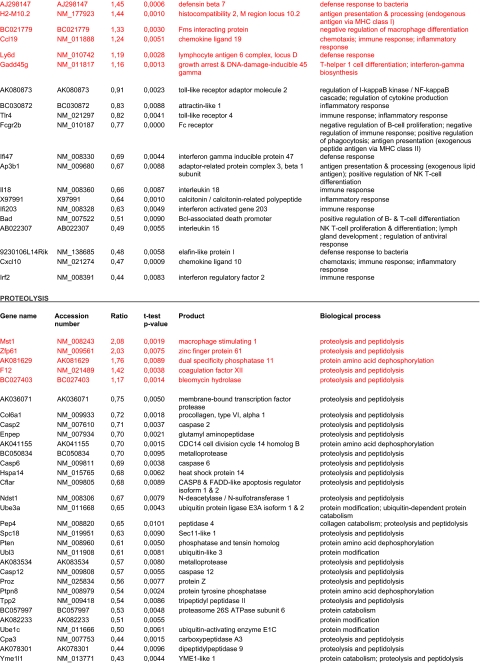
Significantly up- and down-regulated tumor tissue genes controlling important steps of tumor progression during indomethacin treatment of MCG 101 inoculated mice.
